# Enhancing the early home learning environment through a brief group parenting intervention: study protocol for a cluster randomised controlled trial

**DOI:** 10.1186/s12887-016-0610-1

**Published:** 2016-06-02

**Authors:** Jan M. Nicholson, Warren Cann, Jan Matthews, Donna Berthelsen, Obioha C. Ukoumunne, Misel Trajanovska, Shannon K. Bennetts, Tessa Hillgrove, Victoria Hamilton, Elizabeth Westrupp, Naomi J. Hackworth

**Affiliations:** Parenting Research Centre, Melbourne, Australia; Judith Lumley Centre, La Trobe University, 215 Franklin St, Melbourne, 3000 VIC Australia; Murdoch Childrens Research Institute, Melbourne, Australia; School of Early Childhood, Queensland University of Technology, Brisbane, Australia; NIHR CLARHC South West Peninsula (PenCLAHRC), University of Exeter, Exeter, UK; Department of Paediatrics, The University of Melbourne, Melbourne, Australia; The Fred Hollows Foundation, Melbourne, Australia

**Keywords:** Early childhood, Cluster randomised controlled trial, Home learning environment, Parenting group intervention, Playgroups, Home coaching, Socioeconomic disadvantage

## Abstract

**Background:**

The quality of the home learning environment has a significant influence on children’s language and communication skills during the early years with children from disadvantaged families disproportionately affected. This paper describes the protocol and participant baseline characteristics of a community-based effectiveness study. It evaluates the effects of *‘smalltalk’*, a brief group parenting intervention (with or without home coaching) on the quality of the early childhood home learning environment.

**Methods/design:**

The study comprises two cluster randomised controlled superiority trials (one for infants and one for toddlers) designed and conducted in parallel. In 20 local government areas (LGAs) in Victoria, Australia, six locations (clusters) were randomised to one of three conditions: standard care (control); *smalltalk group-only* program; or *smalltalk plus* (group program plus home coaching). Programs were delivered to parents experiencing socioeconomic disadvantage through two existing age-based services, the maternal and child health service (infant program, ages 6–12 months), and facilitated playgroups (toddler program, ages 12–36 months). Outcomes were assessed by parent report and direct observation at baseline (0 weeks), post-intervention (12 weeks) and follow-up (32 weeks). Primary outcomes were parent verbal responsivity and home activities with child at 32 weeks. Secondary outcomes included parenting confidence, parent wellbeing and children’s communication, socio-emotional and general development skills. Analyses will use intention-to-treat random effects (“multilevel”) models to account for clustering.

**Recruitment and baseline data:**

Across the 20 LGAs, 986 parents of infants and 1200 parents of toddlers enrolled and completed baseline measures. Eighty four percent of families demonstrated one or more of the targeted risk factors for poor child development (low income; receives government benefits; single, socially isolated or young parent; culturally or linguistically diverse background).

**Discussion:**

This study will provide unique data on the effectiveness of a brief group parenting intervention for enhancing the early home learning environment of young children from disadvantaged families. It will also provide evidence of the extent to which additional one-on-one support is required to achieve change and whether there are greater benefits when delivered in the 1st year of life or later. The program has been designed for scale-up across existing early childhood services if proven effective.

**Trial registration:**

8 September 2011; ACTRN12611000965909.

## Background

The skills acquired in the early years of life are key foundations for a successful transition to kindergarten and school, and strongly influenced by the quality of the home learning environment [1–3]. Impoverished early life home environments are associated with a range of poorer developmental outcomes [4, 5]. Large-scale community interventions to improve the quality of young children’s home learning environments have seldom been rigorously evaluated [6, 7]. This paper describes a large community-based effectiveness study designed to address this gap. The study comprises two cluster randomised controlled trials (RCTs), one for infants and one for toddlers. The trials are conducted in parallel and evaluate the effects on home learning environment of a brief group parenting intervention for disadvantaged families. The intervention has been designed for future use in early childhood services, and the study additionally seeks to address implementation questions regarding the optimal timing and amount of individual support required for change.

Twenty-three percent of Australian children lack key early learning skills when they commence school [8]. Socioeconomic disparities in learning and development are evident from birth and persist across childhood [9]. To narrow these gaps, programs are needed that successfully engage disadvantaged families and are effective in changing the modifiable mechanisms that underpin socioeconomic differences. As described below, the daily interactions that occur between parents and children are one such mechanism.

### Parenting and the home learning environment

A home environment rich in language and age-appropriate stimulating play activities has a strong positive impact on children’s development in early childhood [3, 10–13]. Responsive interactions characterised by parental sensitivity, warmth and cognitive stimulation promote neurological development and the acquisition of cognitive and language skills [11, 14–18]. Parenting sensitivity refers to parents’ attunement to their child’s cues, emotions, interests, and capabilities in ways that balance the child’s need for support with the need for autonomy. Parenting warmth refers to parents’ expressions of affection and respect toward their children supporting skills for learning such as mastery, security, autonomy, and self-efficacy. Cognitive stimulation refers to parental efforts to enrich their children’s cognitive and language development through language-rich interactions and activities that promote learning.

Early childhood parent–child interactions have been shown to mediate the effects of family socioeconomic disadvantage on developmental outcomes [19, 20]. For example, parental sensitivity and the provision of cognitively stimulating activities reduce the adverse effects of disadvantage on children’s language and cognitive abilities [12, 21]. Supporting high-quality parenting may therefore be an effective way to mitigate the developmental risks faced by young children from disadvantaged families.

### Early childhood parenting interventions for disadvantaged families

Parenting interventions can be effective in supporting parents to provide a rich home learning environment for their young children [6, 22]. Intensive home visiting interventions have shown variable degrees of success [23], with greater improvements reported for high fidelity programs involving frequent visits by professionally-qualified staff [24, 25]. These approaches have limited potential for large scale provision, as they are costly to deliver and have reported difficulties engaging and retaining families over time [23, 26].

While there is a clear need for interventions that can be provided on a wider scale, only a few studies have examined the efficacy of brief programs addressing the quality of the home learning environment [27]. Two studies [28, 29] found that a structured home-based curriculum was associated with increases in responsive parenting behaviours, greater use of home learning strategies and improved infant social and cognitive skills 3 to 6 months post intervention. Home-based approaches are costly to provide and it is unknown whether similar effects could be obtained via community-based group programs. It is possible that brief home-based intervention provided as an add-on to group programs may enhance potential outcomes through the reinforcement of program content and provision of additional individual support and appropriate referral [30], but this has yet to be evaluated using an appropriate controlled design.

### The current study

In Australia, no large-scale experimental studies have evaluated the effectiveness of brief parenting interventions that seek to enrich the early home learning environment of children from disadvantaged families. The current research was commissioned by the State Government of Victoria to address this research gap. The goal was to conduct a large-scale effectiveness study to determine whether a brief group parenting intervention (the *smalltalk* program) delivered within existing community services could improve the capacity of parents experiencing social and economic disadvantage to provide a rich home learning environment to their young children. This presented a unique opportunity to embed a major service development initiative within a rigorous scientific framework and to build knowledge that would guide future early childhood policy and services.

### Development of the *Smalltalk* programs

The *smalltalk* programs were designed for delivery within the existing structures and human resources of the Australian early childhood sector. Five pragmatic and scientific criteria guided program design: evidence-informed intervention strategies; developmental appropriateness; content able to be delivered reliably and proficiently by early childhood workers; compatibility with existing services; and capacity to provide additional individualised support. The first two of these criteria are described next.

#### Developmentally appropriate, evidence-informed content

*Smalltalk* employed active skills training to increase parent behaviours that would promote children’s development of language and communication skills [13, 31]. Targeted parent behaviours (quality parent–child interactions and provision of a stimulating home learning environment) are defined in Table 1. To support the maintenance of these behaviours, information was provided about self-care, having confidence in one’s parenting skills and building connections with other parents and relevant services.

**Table 1:** *smalltalk* Program Content and Operational Definitions

*Key Parenting Strategies (active skills training in-session and exemplified in DVDs)*
1. Quality parent–child interactions: Responsive interactions characterised by parental sensitivity, warmth and cognitive stimulation
• Tuning in: refers to moments when the parent is fully focussed on what the child is doing, saying and possibly feeling. This creates the opportunity for the parent to be sensitive and responsive to the child’s needs.
• Following the child’s lead: involves paying attention to and building on the child’s interests. This provides opportunities for teachable moments
• Listening and talking more: involves increasing exposure to language (both the frequency and variety of words) in a way that promotes ‘conversation’ (e.g., interactive turn-taking that involves both listening and talking). This is a powerful driver of language development from a very young age.
• Using teachable moments: involve capitalising on everyday opportunities for learning. Children are most open to learning when they are interested in something. A teachable moment arises when a parent encourages a child to extend their knowledge or experience of something with simple comments and questions (e.g., “Yes, it’s a car – what colour is that car?”).
• Being warm and gentle: relates to the tone or quality of the interaction. The expression of affection and acceptance strengthens the relationship between parent and child and has powerful effects on child development and wellbeing.
2. Stimulating home learning environment: An environment rich in language and age-appropriate play activities
• Shared reading: a dialogic (shared) approach to reading that is interactional and relationship-building and promotes the use of both book and non-book literacy resources. Where parents have low literacy themselves, they are encouraged to ‘tell a story’ based on the pictures.
• Learning through everyday routines: predictable, positive daily routines that help children feel secure and provide a daily ‘infrastructure’ for parent–child interactions that promote learning and development (e.g., a bedtime routine that involves reading to children).
• Supporting children’s play: provision of developmentally appropriate play objects and activities essential for child development. Emphasis is given to the use of inexpensive, safe household objects that make excellent toys for learning.
• Using community resources: involves introducing parents to activities and resources in the community such as libraries and toy libraries.
• Monitoring use of media: emphasis is given to choosing age appropriate programs and limiting exposure to advertising and ‘background’ television (e.g., television that is on in the background, which interrupts and distracts children from their activities).
Supporting Information Provided on strategies to build parents’:
• Personal agency: building confidence, efficacy and reflective practice around parenting • Self-care: enhancing/maintaining wellbeing, accessing practical, emotional & informational support, stress management • Community connectedness: increasing parental awareness of and ability to access needed services, being supported by and involved with their community

Children’s developmental skills undergo considerable, rapid development across the first 3 years of life. Approaches for promoting, reinforcing and extending these skills change accordingly. Two versions of the *smalltalk* program were developed: one for parents of infants (6–12 months) and one for parents of toddlers (aged 12–36 months). Key intervention strategies remained consistent across the two formats but different age-appropriate examples were used.

#### The service context

Government-funded programs in the state of Victoria are provided free and universally to disadvantaged families with young children through two key community services—the maternal and child health service and facilitated playgroups. Both services have a policy focus on the enhancement of early child development and offer group programs to parents. Program delivery is coordinated by local government authorities (i.e. councils), either directly or in partnership with community organisations. The maternal and child health service has its highest rates of participation by parents of infants, declining after 12 months of age [32]. Facilitated playgroups are designed to enhance toddlers’ skills through structured play activities and to support parents in their parenting role [33, 34].

Session timing and the methods of instruction employed in the *smalltalk* groups were tailored to these contexts and the skills of existing staff. For the parents of infants, the intervention was structured as a weekly parent education group, established for the purpose of delivering the *smalltalk* content. For the parents of toddlers, *smalltalk* content was delivered via incidental teaching methods within weekly playgroup sessions structured around play activities.

An additional home-based component was developed (*‘smalltalk plus’*) to address concerns that parents facing multiple sources of socio-economic disadvantage may struggle to achieve and maintain behaviour change in the absence of individualised support [35]. It comprised a DVD-based intervention delivered in a series of home visits by a coach as an adjunct to group participation. The narrated DVD provided video modelling of strategies discussed in the group sessions. The DVD prompted the coach to guide the parent through practicing each strategy and to videotape the practice for review and goal setting.

## Aims and hypotheses

The aim of this study was to conduct two parallel cluster RCTs to evaluate the effectiveness of the *smalltalk* and *smalltalk plus* programs with parents from economically and socially disadvantaged circumstances. The RCTs were conducted with parents of infants aged 6 to 12 months and toddlers aged 12 to 36 months respectively. The *smalltalk* programs sought to: (i) improve the quality of parent–child interactions and the home learning environment (primary outcomes, parent focussed) (ii) improve parenting confidence, parents’ wellbeing and community connectedness (secondary outcomes, parent focussed); and consequently (iii) improve children’s early communication, socio-emotional and general developmental skills (secondary outcomes, child focussed).

We hypothesised that in both the infant and toddler trials, families who received the *smalltalk group only* and *smalltalk plus* interventions would show greater improvements in primary outcomes (parent verbal responsivity, home activities with the child at 32-week assessment) and secondary outcomes (parent-reported and directly observed parent–child interactions; the home literacy environment and household disorganisation; parent wellbeing, self-efficacy and community connectedness; and directly observed and parent reported child communication skills) compared to parents who received the *standard* (control) program. In the absence of prior evidence regarding differential outcomes by child age, we made no hypotheses regarding differences in program effectiveness for the infant versus toddler samples.

## Methods and design

### Approval and registration

Ethics approval and permission to conduct the research were obtained from the Victorian Government Department of Health Human Research Ethics Committee (HREC08/10) and the Department of Education and Early Childhood Research Committee. The study is registered as a cluster randomised controlled trial with the Australian New Zealand Clinical Trials Registry (ACTRN 1261 1000965909; Registration date 8 September 2011).

### Design

The study design comprises two cluster RCTs conducted in parallel, one in the maternal and child health service (for parents of infants) and the other in the facilitated playgroup service (for parents of toddlers). The study was conceptualised as an effectiveness trial [36] designed to assess program outcomes as delivered under real-world conditions. It has been implemented and reported in accordance with the requirements of the CONSORT statement for cluster RCTs [37].

**Fig. 1:**
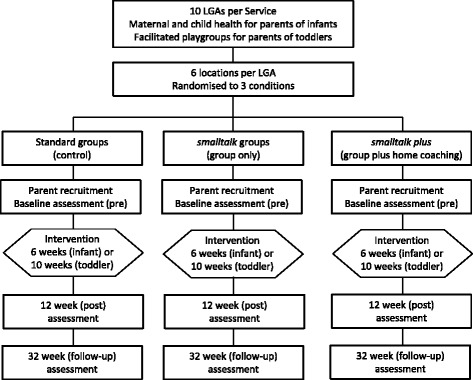
Representation of study design

In each RCT, there were three trial arms (intervention conditions): standard, *smalltalk group-only, smalltalk plus*. Clusters were randomised to condition (1:1:1 allocation ratio), stratified by LGA. Clusters were the geographical location where group programs were to be delivered. Approximately six locations were randomised in each LGA to deliver one of the three programs: standard, *smalltalk group-only,* or *smalltalk plus* programs. Parents were allocated to the location nearest to their residential address and received the intervention delivered by that location. Figure 1 is a diagrammatic representation of the study design for each RCT.

### Site recruitment

The trial was designed to be implemented within funding by the state government with a goal of program delivery to 2000 parent–child dyads across a 2-year period. As part of their service agreements, each of the participating LGAs (10 providing infant programs and 10 providing toddler programs) were funded to recruit and provide programs to 100 parent–child dyads. LGAs were also funded to appoint a site coordinator to oversee recruitment, staff employment, service delivery and reporting.

Twenty LGAs were recruited in metropolitan and rural areas as follows. All 79 LGAs in the state of Victoria were informed about the study through a letter of introduction to Chief Executive Officers, followed by briefings in each administrative region. Meetings with service managers were held as requested, and interested LGAs were invited to apply to participate. Applications were accepted from LGAs that met the following criteria: evidence from administrative data of significant levels of socioeconomic disadvantage in the community; prior successful collaboration with external agencies; willingness to adhere to the design and reporting requirements of the research trial; and experience and capacity to deliver parent groups or facilitated playgroups.

### Allocation

Cluster randomisation of locations was chosen to reduce the potential for cross-condition contamination arising from parents gaining exposure to another condition through others in their immediate community. Additionally, staff were only trained in one of the three program conditions.

Allocation of locations was stratified by LGA using block randomisation with a fixed block size of 3. Locations were allocated in the order that they were consented, in blocks of 3 to maintain blinding during the recruitment of locations. Randomisation was performed by a biostatistician (OU) who was unaware of the identities of the locations and played no role in the recruitment of locations or parents. Researchers involved in parent recruitment and baseline assessment were blind to the trial arm status of the locations, thus, allocation concealment was ensured.

### Intervention delivery

#### Smalltalk program development and content

Program content, methods of delivery and staff training were developed through extensive consultation and a co-production process. In 2010, two one-day forums were conducted with practitioners and service managers to seek input on program content, strategies for engaging disadvantaged families and potential logistic issues. From April to September 2010, members of the research team attended weekly sessions of two existing facilitated playgroups and undertook home visits with a subgroup of families. Parents were asked for feedback on the program content, with particular attention to the way the ideas were expressed, the language used and examples given. Facilitators provided feedback on program content, how it could be used, and the training and resources needed. Finalised program content and staff training processes were then fully field tested in four LGAs from September to December 2010 with the parents (*n* = 39) and staff (*n* = 4) participating in one infant and three toddler groups.

Program content focussed on building parents’ use of 10 daily parenting strategies (summarised in Table 1). Parents were provided with information and active skills training in 5 strategies for enhancing the quality parent–child interactions (e.g., parent responsiveness; positive verbal exchanges where parents respond to and build on the child’s interests) and 5 strategies for providing a stimulating home learning environment (e.g., use of books and toys to extend the child’s developing skills; the provision of daily activities and routines that are language- and literacy-rich). Information was also provided about the importance of looking after oneself (parental self-care), having confidence in one’s parenting skills (personal agency) and building connections with individuals and services in the local community (community connectedness).

#### Program delivery formats—infants

The infant program comprised 6 weekly 2-hour group parenting sessions, designed for attendance by 6 or more parents and their infants. Parents allocated to the active intervention (*smalltalk group-only, smalltalk plus*) received a parent DVD and printed resources illustrating the program’s key parenting strategies (Table 1). Facilitators introduced and guided the practice of the strategies in the group, and assisted parents to plan and report on their use of the strategies at home.

Parents allocated to the *smalltalk plus* program received the group program plus six 60-min individual home visits from an early childhood-qualified ‘home coach’. Sessions were structured around a narrated DVD to maximise program fidelity. The DVD contained filmed exemplars of the intervention strategies and guided the activities for the session. Parents were videotaped practicing the strategies with their child and the footage was jointly reviewed for feedback and goal setting. The DVD included scenes of the program’s strategies being used well and scenes that illustrated missed opportunities for using these strategies.

For parents allocated to the *standard condition*, group sessions focussed on issues relevant to parenting a 6–12 month old infant (e.g. feeding, sleeping, safety, exercise, and behaviour). No elements of the *smalltalk* program were discussed.

#### Program delivery formats—toddlers

The toddler program comprised ten 2-h weekly facilitated playgroup sessions. These were designed for attendance by 10–15 parents and their children and offered in four terms corresponding to the school calendar. Parents allocated to the active intervention (*smalltalk group-only, smalltalk plus)* received a parent DVD and printed resources. They were introduced to the *smalltalk* program content during their first term of attending the facilitated playgroup. Using incidental teaching methods, facilitators discussed the parenting strategies one-on-one or in small groups, structured play activities to provide practice of the strategies, and assisted parents to plan and report on their use of the strategies at home. At the end of the 10 week program parents could remain in the playgroup but were not directly targeted by the playgroup facilitator for incidental teaching activities.

Parents allocated to the *smalltalk plus* condition received the group program plus six 60-min individual home visits from an early childhood-qualified ‘home coach’. Sessions were structured in the same way as for the infant home coaching program, directed by a narrated DVD.

Parents allocated to the *standard condition* attended playgroups conducted according to the objectives and activities of current facilitated playgroups in Victoria, with no *smalltalk* program content.

### Facilitator training and support

*Smalltalk* was designed for delivery by existing early childhood staff. Facilitators and home coaches were employed by the LGAs and received standardised training from the research team. Of the 109 staff who were trained to deliver programs almost all were female (*n* = 108), aged from 23 to 59 years (mean = 42). Fourteen percent had post-graduate qualifications, 28 % had a bachelors degree and 56 % had post-secondary vocational qualifications. Qualifications were in the fields of community services (46 %), education (29 %), health (12 %), or other (13 %). On average staff had 15.5 years of experience in the early childhood community sector (range 0 to 37 years).

All staff received half- or full-day training in group facilitation (for infant and toddler groups respectively). *Smalltalk* facilitators and home coaches received an additional 2–3 days training in the program content and delivery procedures. Training resources included a comprehensive training manual, tip sheets, activity sheets and wall posters illustrating the intervention strategies. Home coaches also received session planning guides, record keeping books and the home coaching DVD. The research team offered post-training support by email, telephone and text messaging to address any arising issues.

### Participant recruitment and eligibility criteria

LGAs were responsible for recruitment of families into the trial. Eligibility criteria were: living within the geographical boundaries of a trial location; having at least one child in the age range for the offered program (6–12 months for infant programs and 12–36 months for toddler programs); and evidence of at least one identifiable risk factor for poor child development, including low family income; receipt of government benefits or holder of a Health Care Card (provided for low income families); single, socially isolated or young parent (≤25 years); and culturally and linguistically diverse background. Parents were not eligible for participation if they were aged less than 18 years; did not speak English; were involved with child protection services; already received in-home support; or were deemed to require more intensive services.

Information on inclusion and exclusion criteria was available through each LGA’s maternal and child health administrative database. LGAs were encouraged to identify potential participants via case finding (e.g. searches of the database for eligible families) and rolling recruitment (e.g. assessing families for eligibility at routine child health checks; outreach through relevant community services). Staff in the LGAs were provided with scripts for recruiting participants, and promotional brochures and flyers to enhance the visibility of the study.

Participants identified as eligible for the study were contacted by the LGA site coordinator who explained the research and obtained verbal consent for participation and for their contact details to be sent to the research team. Verbal consent was repeated at the start of the baseline telephone interview and full written consent was obtained at the baseline visit to collect in-home observation data.

Based on previous experience with similar populations [38, 39], we aimed to retain at least 85 % of the enrolled sample to follow-up (T = 32 weeks). Strategies to support participation included a $50AUD payment and a children’s book provided at each time-point (pre, 12 weeks and 32 weeks) to parents who completed the assessments in full. Payments were reduced to $20AUD for parents who provided partial data. Participants were not paid for attending program sessions.

### Measures

Multi-method data collection occurred at three main time points: baseline (0 weeks); post-intervention (12 weeks); and follow-up (32 weeks) (see Fig. 1). Participant characteristics and individual-level outcomes data were collected by parent report and direct observation. Process data were collected by administrative records and staff report.

- *Parent-report data* were collected via computer assisted telephone interviews (CATI) to allow inclusion of parents with low literacy. These were conducted at pre, post (12 weeks), and follow-up (32 weeks) by trained interviewers, independent of the research team and blinded to participant allocation. As summarised in Table 2, the CATI included a number of brief, validated measures of parent and child outcomes (all time points), parent, child and family characteristics (baseline only), and ratings of satisfaction with the program and barriers to participation (post only; asked at the end of the interview to avoid unblinding the interviewer during the collection of outcomes data). Included measures were primarily sourced from the Longitudinal Study of Australian Children [40] or other evaluation studies [39]. Parents also completed a pencil and paper version of the Communicative Development Inventory (CDI) during the home visit (see below), or over the telephone with a research staff member.

**Table 2:** Summary of Study Measures

Variable	Measure	Data collection	
		Method^a^	Collected^b^
*Primary outcomes*			
Parental verbal responsivity	StimQ-T [47]: 4 items on a 4-point scale E.g. “Talk about the day while your child is eating”, summed to produce a total score between 4 and 16.	CATI	Pre, post, FU
Home learning activities	Home activities with child: 5 items on a 4-point scale assessing parental engagement of child in home activities that stimulate development [48] E.g. “Read books to your child”, summed to produce a total score between 4 and 20.	CATI	Pre, post, FU
*Secondary outcomes*			
*Parent–child interactions*			
Parental warmth	Warmth: 6 items on a 5-point scale scale from the Longitudinal Study of Australian Children (LSAC) [40], “Thinking about the last 6 months, how often do you…” E.g. “Hug or hold your child for no reason”, summed to produce a total score between 6 and 30.	CATI	Pre, post, FU
Parental irritability	Irritability: 5 items on a 5-point scale from LSAC [40], “Thinking about the last 4 weeks, how often have you…” E.g. “Lost your temper with your child”, summed to produce a total score between 5 and 25.	CATI	Pre, post, FU
Parent interactions	Indicator of Parent – Child Interaction: Caregiver interactions coded as ‘facilitators’ or ‘interrupters’ [42] E.g. “conveys acceptance and warmth” and “uses criticism or harsh voice”. Interactions are rated on a 4-point scale of relative frequency, from 0 = never to 3 = often/consistently.	Observed	Pre, post, FU
*Home environment*			
Home literacy	Home Literacy Environment Scale: 6 items on various scales, [49], E.g. “How many books does your child own?”, summed to produce a total score ranging from 0 to 11.	CATI	Pre, post, FU
Disorganisation	Confusion, Hubbub and Order Scale (CHAOS-SF): 6 items on a yes/no scale [50, 51], E.g. “The atmosphere in our home is calm”, summed to produce a total score ranging from 0 to 4.	CATI	Pre, post, FU
*Parent focussed outcomes*			
Psychosocial distress	Kessler-6 (K6): 6-item psychosocial screener on a 5-point scale assessing emotional distress in the last 4 weeks [52]. “About how often did you feel:” E.g. “nervous”, summed to produce a total score between 0 and 24.	CATI	Pre, post, FU
Wellbeing	SF-12: 12-item health related quality of life [53] on various scales E.g. “How much does your health limit you in climbing several flights of stairs?” and “How much of the time during the past 4 weeks have you felt calm and peaceful?”, producing a Physical Health summary score and a Mental Health summary score.	CATI	Pre, post
Psychological adjustment	I-PANAS-SF: 5-item positive affect subscale on a 5-point scale [54], “Thinking about yourself in the last 4 weeks, about how often did you feel…E.g. “alert?”, summed to produce a total score between 5 and 25.	CATI	Pre, post, FU
Parent confidence	1 item on a 5-point scale, overall efficacy as a parent from LSAC [55], “Overall, as a parent, do you feel that you are…” E.g. “a better than average parent”, producing a score between 1 and 5.	CATI	Pre, post, FU
Parental self-efficacy	4 items on a 5-point scale, infant and toddler versions of parental self-efficacy from LSAC [39], “In general, do you feel that you are…?” E.g. “Very good at keeping your child amused”, summed to produce a total score ranging from 5 to 20.	CATI	Pre, post, FU
Community connectedness	Use of early childhood services: 6 items on a yes/no scale, study-developed to assess past, current or intended use of similar early childhood programs. “Have you or your child ever attended any other services or programs to assist you and your child?” E.g. “early intervention program”.	CATI	Post
	Contact with other parents: 2 items assessing contact with other parents outside the program [39] “Have you had contact with any of the other parents outside the sessions?” and if so, “Do you think this contact will continue?”	CATI	Post
*Child focussed outcomes*			
Communication skills	Ages and Stages Questionnaire (ASQ) Communication subscale [56]: 6 items on a 3-point scale. E.g. “Does your child point to, pat, or try to pick up pictures in a book?” Scored yes = 10, sometimes = 5, not yet = 0; summed to a total score between 0 and 60.	CATI	Pre, post, FU
Vocabulary	MacArthur-Bates Communicative Development Inventory (CDI) [57, 58]. Three age versions of the Short Form vocabulary checklists. Level I, up to 18 months: 89 words the child “understands” or “understands and says” (e.g. “mummy” and “meow”). Level II, 19–30 months: 101 words (e.g. “book” and “finish”) and 1 item assessing use of word combinations. Level III, 31 months and older: 100 words (e.g. “then” and “today”), 12 sentence pairs to evaluate complexity of language use, and 12 yes/no items assessing language comprehension.	Parent-report	Pre, post, FU
	Early Communication Indicator (ECI) [59]: frequency of gestures, vocalisations, single words and multiple words generated for each minute of 6-min play activity. Instances of communication are tallied, with weightings for single words (multiplied by 2) and multiple words (multiplied by 3) to produce a total communication score.	Observed	Pre, post, FU
Socio-emotional skills	ASQ Personal-Social subscale [56]: 6 items on a 3-point scale, E.g., “Does your child play with a doll or stuffed animal by hugging it?” Scored yes = 10, sometimes = 5, not yet = 0; summed to a total score 0–60.	CATI	Pre, post, FU
General development	ASQ Fine Motor subscale: [56] 6 items on a 3-point scale, E.g. “Does your child stack three small blocks or toys on top of each other by herself?” Scored yes = 10, sometimes = 5, not yet = 0; summed to a total score 0–60.	CATI	Pre, post, FU
*Process measures*			
Parent engagement	Attendance checklist and facilitator ratings of parent engagement [39] E.g. “Parent engagement with other parents” on a 5-point scale from 1 = did not talk with other parents to 5 = talked to many other parents.	Staff ratings	Each session
Program delivery	Program quality and integrity: 6 items rated by facilitators [39], E.g. “Level of rapport and engagement established” on a 5-point scale from 1 = much less than expected to 5 = much better than expected.	Staff ratings	Each session
Program intensity	Study designed, facilitator checklist of content coverage.	Staff ratings	Each session
Parent satisfaction	6 items on a 4-point scale assessing parents satisfaction with the program, staff and knowledge gains [38] E.g. “Overall, how satisfied or dissatisfied were you with the program?”	CATI	Post
Participation barriers	13 items on a yes/no scale assessing barriers to program participation [38] E.g. “difficulties relating to other parents”, “work commitments”.	CATI	Post
Staff training	Ratings of program quality (2 items: clarity, usefulness), preparedness to deliver it (3 items: confidence, well-prepared, difficulty), and satisfaction with training (5 items: clarity, usefulness of materials/presentation) on 5-point scales.	Staff ratings	After training
Staff self-assessment	6 skills for program delivery with the target population, E.g. “Identifying specific needs of families” on a 5-point scale from 1 = ‘no level of skill/knowledge in the area’ to 5 = ‘advanced level of skill/knowledge’.	Staff ratings	Before, after training
*Covariates*			
Demographics	Parent age, ethnicity, language spoken, education, income, employment status family structure and size	CATI	Pre
Child characteristics	Child age, ethnicity, general health, disability, special health services, birth weight	CATI	Pre
Child temperament	4 items on 3-point and 4-point scales, modified version of the NEILS Scales of Developmental Competency [38, 60], E.g. “Would you say that your child is easy to manage, sometimes hard to manage or often hard to manage?”, scores ranging from 4 to 12.	CATI	Pre, post, FU
Parent depression	Single item yes/no rating from LSAC, “In the past year, have you had 2 weeks or more during which you felt sad, blue or depressed, or lost pleasure in the things that you usually cared about or enjoyed?” (0 = no; 1 = yes).	CATI	Pre
Parent coping	Single item on a 5-point scale from LSAC, “How well do you think you are coping?” producing a score 0–5.	CATI	Pre, post, FU
Stressful life events	List of Threatening Experiences (LTE-Q): 7-item yes/no list of life adverse life events in last 12 months, [61] E.g. “You had a major financial difficulty”, producing a total score between 0 and 7.	CATI	Pre, post, FU

^a^CATI = Computer Assisted Telephone Interview

^b^Pre = completed prior to program commencement; post = completed after last program session, approximately 12 weeks after pre; follow-up (FU) = completed 32 weeks after pre
- *Observational data* were collected in the parent’s home by trained and accredited research staff or home coaches, at pre, post and follow-up (Table 2). Data were collected according to standardised protocols for two ‘Individual Growth and Development Indicators’ assessment procedures (described below) [41]. These assessments provide good capture of the parent and child outcomes targeted by the *smalltalk* programs, have been validated for use with parents of children aged 2–42 months, and have demonstrated reliability and validity among disadvantaged populations [41, 42].
- The Indicator of Parent–Child Interaction (IPCI) assesses the extent to which parents respond to their child in ways that promote positive communication and social-emotional behaviours during 8–10 min of: free play (4 min); looking at books (2 min); a dressing task (2 min); and a distraction task (2 min; only for children 12 months and older). Interactions were videotaped for later frequency coding. Six parent behaviours (four ‘facilitating’ and two ‘interrupting’ behaviours) were tallied for each task and then an overall rating was made for all tasks combined (behaviours coded as ‘0 = never occurs’ to ‘3 = occurs often). Scores are the frequencies for each behaviour separately and summed for the facilitators (warmth and acceptance; descriptive language; follows child’s lead; maintains child’s interest) and interrupters (harsh comments; restrictions) [42].
- The Early Communication Indicator (ECI) assesses four child communication skills (use of gestures, vocalisations, single words and multiple word utterances), demonstrated during a 6-min parent–child play activity with standardised toys. Later coding involved tallying the number of skills demonstrated per minute. The final score was a weighted sum that gives greater weight to more advanced communication skills (a weighting of two for single words and three for multiple word utterances) and allows for comparisons between children of different ages [41].
- Coding was undertaken by two accredited, expert coders according to standardised protocols. Coders were blind to the study design, participant allocation and the data collection time point. Twenty percent of observations were independently coded by both assessors to determine inter-rater reliability (percent agreement).
- Due to the high costs of coding, an initial 600 observations (100 participants each from the maternal child health and playgroups services assessed at three time points) were randomly selected, stratified by location (to preserve the clustered design) for coding.
  - *Administrative records:* Numbers of parents who expressed interest, were recruited and retained at each phase of the study were collected via administrative reporting procedures and tracking databases.
  - *Program staff ratings:* Program fidelity, program quality, participant attendance and participant engagement in sessions were rated using standardised checklists by facilitators and home coaches at the end of each group or home coaching session (see Table 2). Reliability was checked by comparison with the independent ratings by research members attending a sample of group sessions.

### Sample size

Our target was to recruit 22 locations (clusters) and 308 parent–child dyads (14 parent–child dyads from each location) in each of the three arms (*smalltalk plus*; *smalltalk group-only*; *control*) for *each* RCT (infant and toddler). The intended sample size is large enough to detect a difference of 0.3 standard deviation units (effect size) between any two trial arms within each of the infant and toddler trials with 90 % power at the 5 % level of significance, allowing for an intra-cluster (intra-location) correlation coefficient of 0.01 and 15 % loss to follow-up at the parent–child dyad level.

### Data analyses

Baseline characteristics will be summarised by trial arm (intervention condition) using means and standard deviations for continuous data and frequencies and percentages for categorical data. For all hypotheses, individual-level outcomes will be compared between the *smalltalk group-only* and control arms and between the *smalltalk plus* and control arms at post-intervention (12 weeks) and follow-up (32 weeks), separately for each of the infant and toddler programs. These comparisons will be based on the intention-to-treat principle analysing the parent–child dyads according to the trial arm their location (cluster) was randomised to without regard to the amount of intervention actually received. Random effects (“multilevel”) linear regression models [43] will be used to compare continuous outcomes between the trial arms. Marginal logistic regression models using Generalised Estimating Equations (GEEs) with information sandwich (“robust”) estimates of standard error will be used to compare binary outcomes. An exchangeable correlation structure will be specified for the GEE method. The random effects model and GEE method allow for correlation between the responses of dyads from the same location cluster. Crude (unadjusted) estimates (mean difference and odds ratio) and estimates that are adjusted for the baseline score of the outcome, child age and gender, single parent family status, language other than English spoken at home, mother 25 years of age or younger, education below year 12, and unemployment status will be reported.

## Trial status and baseline data

Site recruitment occurred in two stages in mid-2010 and early 2011. Staff training, parent recruitment and baseline assessments commenced in 2011. Programs were delivered across seven school terms from February 2011 to October 2012. Follow-up data collection was completed by March 2013. Findings from preliminary data analyses (partial data only) have been presented to the government funders to inform service planning [44]. This report has not been publically released. Analyses of outcomes, process and baseline data are ongoing. The state government has subsequently funded the Parenting Research Centre in Melbourne to oversee the integration of *smalltalk* programs into usual practice across the state. In partnership with the state government, funding has also been obtained to assess the maintenance of program effects on parent and child outcomes when the children are aged 7–8 years (NHMRC Partnership Grant Application APP1076857).

### Recruitment and participant characteristics

**Fig. 2:**
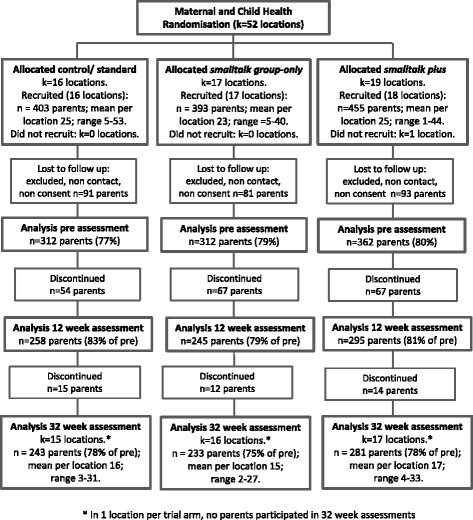
Participant flow in the infant trial

**Fig. 3:**
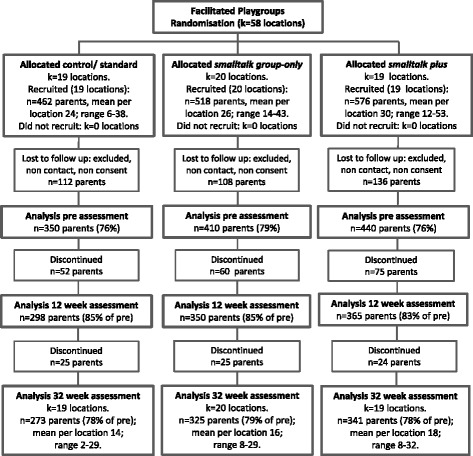
Participant flow in the toddler trial

The study was successful in recruiting twenty LGAs (110 locations) to participate in the study. Ten LGAs ran infant programs and 10 ran toddler programs, with a total of 389 programs provided from 109 locations (clusters): 51 in the infant trial; 58 in the toddler trial. Figures 2 and 3 present the participant flow for each RCT. Across the trial arms, 76–80 % of those recruited were able to be recontacted, gave full study consent and provided baseline data.

Participants (see Table 3) assessed at baseline were 2186 parents: 986 were parents of infants (aged 6–12 months) enrolled through the maternal and child health service and 1200 were parents of toddlers (aged 12–36 months) enrolled through the facilitated playgroup service. Of those enrolled, 86 % (*n* = 1890) attended at least one group session. Retention to follow-up was excellent. Data were provided at 32-week follow-up by 75–78 % of parents in the infant trial (see Fig. 2) and 78–79 % of parents in the toddler trial (see Fig. 3).

**Table 3:** Baseline characteristics of recruited samples in the maternal and child health and facilitated playgroups RCTs

Characteristics	Maternal and Child Health (infant) RCT				Facilitated Playgroups (toddler) RCT			
	standard *N* = 312	*smalltalk* group-only *N* =312	*smalltalk plus* *N* = 362	Total *N* = 986	standard *N* = 350	*smalltalk* group-only *N* = 410	*smalltalk plus* *N* = 440	Total *N* = 1200
Child								
Female, n (%)	164 (52.6)	144 (46.2)	182 (50.3)	490 (49.7)	169 (48.3)	210 (51.3)	240 (54.3)	619 (51.5)
Child age in months, mean (SD)	7.9 (2.4)	8.1 (2.2)	8.0 (2.2)	8.0 (2.3)	21.7 (7.5)	22.3 (7.2)	22.8 (7.1)	22.33 (7.2)
Indigenous, n (%)	7 (2.3)	8 (2.6)	10 (2.8)	25 (2.5)	3 (0.9)	9 (2.2)	8 (1.8)	20 (1.7)
Parent								
Male, n (%)	4 (1.3)	4 (1.3)	3 (0.8)	11 (1.1)	19 (5.4)	19 (4.7)	13 (2.9)	51 (4.3)
Parents' age in years, mean (SD)	30.5 (5.1)	31.2 (5.7)	31.1 (6.0)	30.9 (5.6)	33.3 (5.9)	33.5 (5.8)	33.2 (6.2)	33.33 (6.0)
Aged ≤ 25 years, n (%)	60 (19.2)	57 (18.3)	70 (19.3)	187 (19.0)	34 (9.7)	39 (9.5)	41 (9.3)	114 (9.5)
Indigenous, n (%)	5 (1.6)	3 (1.0)	5 (1.4)	13 (1.3)	0 (0.0)	6 (1.5)	6 (1.4)	12 (1.0)
Single parent family, n (%)	39 (12.5)	41 (13.1)	45 (12.4)	125 (12.7)	48 (13.7)	38 (9.3)	50 (11.3)	136 (11.3)
Born overseas, n (%)	50 (16.0)	38 (12.2)	48 (13.3)	136 (13.8)	122 (34.9)	128 (31.3)	137 (31.0)	387 (32.2)
Non-English Language, n (%)	41 (13.1)	34 (10.9)	50 (13.8)	125 (12.7)	120 (34.3)	146 (35.7)	130 (29.4)	396 (33.0)
No parent employed, n (%)	32 (10.3)	47 (15.1)	58 (16.0)	137 (13.9)	47 (13.4)	51 (12.4)	64 (14.6)	162 (13.5)
Did not complete high school (year 12), n (%)	41 (13.1)	47 (15.1)	57 (15.8)	145 (14.7)	42 (12.0)	47 (11.5)	50 (11.3)	139 (11.6)
Main income from pension/benefit, n (%)	50 (16.1)	67 (21.5)	69 (19.1)	186 (18.9)	69 (19.7)	65 (15.9)	77 (17.4)	211 (17.6)
Low income (≤$36,400 AUD), n (%)	58 (19.3)	69 (22.8)	75 (21.5)	202 (21.2)	79 (23.8)	80 (20.4)	90 (21.0)	249 (21.6)

Parents in the infant RCT were mostly biological mothers (99 %), with a mean age of 31 years. Thirteen percent were single parents and 14 % were born outside Australia. Parents in the toddler RCT were also mostly biological mothers (96 %), with a mean age of 33 years. Eleven percent were single parents and one-third (32 %) were born outside Australia. Across the two RCTs, very few participating parents or children identified as Indigenous (1 and 2 % respectively). Around 5 % came from households where there was no parent in paid employment, and around 20 % had a very low income or received their main income from government benefits. As shown in Table 3 there was no evidence of baseline differences in the characteristics of parents by group allocation.

The study was successful in recruiting families experiencing socioeconomic disadvantage. At baseline, 84 % of participating families displayed one or more of the following risk factors for poor child development: young parent, single parent, language other than English spoken at home, low parental education, low family income, receipt of government benefits, low parenting self-efficacy, or parent psychological distress. The study was also successful in attracting families experiencing multiple challenges. Over half the families reported two or more risk factors and approximately 20 % reported four or more risk factors.

## Discussion

This cluster randomised controlled trial is the largest experimental study undertaken in Australia to improve the quality of the home learning environment during a child’s formative years. The study seeks to determine whether a brief group parenting intervention can assist parents from socially and economically disadvantaged circumstances to enhance the home learning environment of their 6–36 month old children. By concurrently undertaking two independent cluster RCTs, the study will provide new information regarding the relative effectiveness of intervening during infancy compared to the toddler years.

This study will also provide insight into the relative benefits of adding an individualised, highly structured home-based component to the group intervention. Only one study to our knowledge has examined a home-based addition to a group parenting intervention involving 10 home-based sessions delivered by a trained therapist [30, 45]. In a non-controlled pre-post design, Lees and Fergusson [45] reported acceptable recruitment and retention with improved parent and child outcomes. The current study employs a home visiting component that is feasible within the Australian early childhood sector and will provide the first rigorous evidence internationally of the extent to which benefits for this approach exceed those achieved by the group program alone.

The way the intervention was developed and the conduct of the research trial within existing community services, addresses a number of the concerns that are directed at traditional efficacy studies [36, 46]. In particular, it was designed to ensure the trial service delivery conditions were a good match to how the programs would be used in the future. Locally-based services received program funding based on enrolments and were responsible for parent recruitment, staff employment and program scheduling. This ensures that the resulting trial data are relevant to the state government funders and community service providers. Co-production and extensive consultation during program development, further aimed to enhance future uptake of the programs by ensuring end-user acceptability and maximising the sense of program ownership. Early indicators suggest that the program has been successful in attracting families from the target population.

In seeking to design and implement a study that has strong external validity, we have not ignored internal validity and data quality. Strengths of the design include: the collection of observational data in addition to parent self-report; collection of detailed process data to guide future refinements; the use of attention-matched control conditions that reflected the programs currently offered; and the use of a cluster design to minimise cross-condition contamination. A possible weakness is the absence of a fourth trial arm that evaluates the effectiveness of the home-coaching component alone. Home coaching alone was considered unlikely for future implementation. Group-based programs are more efficient to deliver and building social connections was an important policy goal. The results of this trial will provide valuable data of international relevance on a novel approach to enhancing the home learning environment for young children from disadvantaged circumstances, whilst providing practical information to service providers in Australia.

### Abbreviations

AUD, Australian dollars ($1AUD roughly equivalent to $0.70US); ECI, Early Communication Indicator; IPCI, Indicator of Parent–Child Interaction; LGA, Local Government Area; RCT, randomised controlled trial
